# Patient Perceptions Towards Reduction or Avoidance of Opioids After Knee and Hip Arthroplasty: A Cross-Sectional Survey

**DOI:** 10.7759/cureus.83977

**Published:** 2025-05-12

**Authors:** Mansi Patel, Darren Young Shing, Annie George, Dhivya Bhaskaran, Anthony Atalian, Manpreet Walia, Milin Patel, Daniel Tushinski, Anthony Adili, Kamal Bali, Vickas Khanna, Sheila Sprague, Kim Madden, Jason W Busse

**Affiliations:** 1 Division of Orthopedics, McMaster University, Hamilton, CAN; 2 Faculty of Medicine, University of Ottawa, Ottawa, CAN; 3 Temerty Faculty of Medicine, University of Toronto, Toronto, CAN; 4 Faculty of Science, McMaster University, Hamilton, CAN; 5 Faculty of Engineering, McMaster University, Hamilton, CAN; 6 Research Institute, St. Joseph's Healthcare Hamilton, Hamilton, CAN; 7 Department of Health Research Methods, Evidence, and Impact, McMaster University, Hamilton, CAN

**Keywords:** opioid reduction, opioids, opioid-sparing strategies, postoperative pain management, total hip arthroplasty, total knee arthroplasty

## Abstract

Background and objective

Opioid analgesics are routinely prescribed to manage pain after total joint arthroplasty (TJA) but are associated with several adverse effects. There is a scarcity of literature exploring patients’ receptivity, attitudes, and perceptions towards opioid-sparing postoperative protocols. In light of this, we conducted this study to address those gaps in the literature.

Methods

We administered a cross-sectional survey to patients aged 18 years or older who were attending either a preoperative or a postoperative TJA appointment, up to 12 months after surgery. We aimed to determine the proportion of patients who would be open to receiving opioid-free or opioid-reduced postoperative care, identify patient characteristics associated with receptivity, and determine patients’ perceptions regarding the efficacy and safety of opioids. We constructed multivariable logistic regression models to explore features associated with patients’ receptivity to opioid reduction or avoidance.

Results

We approached 200 patients, and 190 returned a complete survey. A quarter of respondents believed that other analgesics were similarly effective or superior to opioids, and 68% perceived that opioids were associated with more side effects than alternatives. Of note, 50% of patients indicated that they would be receptive to reduced opioid use postoperatively. Patients’ receptivity was associated with not using opioids at the time of survey completion [odds ratio (OR): 2.5, 95% confidence interval (CI): 1.04-6.4), and the belief that opioids had more side effects than alternatives (OR: 3.4, 95% CI: 1.5-7.9); 40% of respondents indicated they would be willing to avoid opioid use after surgery, and receptivity was associated with the belief that opioids cause more side effects than alternatives (OR: 4.3, 95% CI: 1.8-11.9) and that non-opioid analgesics are similarly or more effective (OR: 3.4, 95% CI: 1.4-8.3).

Conclusions

Many participants were willing to reduce or avoid the use of postoperative opioids, and receptivity was strongly associated with beliefs regarding the comparative benefits and harms of alternatives. These findings suggest opportunities to reduce the use of opioids after TJA and enhance patient education to address misconceptions.

## Introduction

Total knee arthroplasty (TKA) and total hip arthroplasty (THA) are the most common procedures used to treat end-stage osteoarthritis (OA) unresponsive to conservative care. Approximately 29% of THA and 51% of TKA patients experience moderate to severe acute pain after surgery [1]. Moreover, despite a favourable outcome for most, 25% of patients report chronic pain after TKA and 11% report it after THA [2,3]. Opioid analgesics are often included in postoperative pain management regimens following total joint arthroplasty (TJA) [4]. The liberal use of opioid analgesics in the treatment of pain has contributed to the opioid crisis in North America [5,6]. Canada and the USA lead in the per-person consumption of prescription opioids globally [6]. Specifically, there were 22,828 deaths from opioid toxicity between 2016 and 2021 in Canada, with 6,946 deaths occurring during the COVID-19 pandemic between April 2020 and March 2021 [7]. Thus, there is increasing interest in opportunities to reduce opioid prescribing.

Orthopaedic surgeons are the third-highest opioid prescribers in the USA and are responsible for approximately 60% of opioid prescriptions to opioid-naïve patients [8]. A prospective study of 110 patients undergoing primary THA or TKA at an academic center in the USA reported that THA patients consumed significantly fewer opioids than TKA patients [9]. However, both groups received more opioids (a single prescription for oxycodone 5 mg totaling 84 tablets) than were used [9]. Ongoing research suggests that opioid-free or opioid-reduced postoperative care may represent an opportunity to help mitigate the opioid crisis [10,11].

Opioid-reduced/free multimodal analgesia regimens may include pharmacologic modalities (e.g., peripheral nerve blocks, cyclooxygenase-2 inhibitors, gabapentinoids, local anesthetic infiltration, acetaminophen, NSAIDs) and nonpharmacologic modalities that are considered as adjuncts (e.g., acupuncture, electrotherapy, transcutaneous electrical nerve stimulation)[12]. A prospective cohort study with 386 TKA patients receiving a multimodal protocol combining patient-specific preoperative education, continuous adductor canal block with ropivacaine, and self-directed postoperative rehabilitation, found that 86.3% of patients required 10 or fewer opioid pills through 12 weeks postoperatively [13]. Another retrospective study, implementing an opioid-sparing pain management protocol for THA, found a significant reduction in inpatient and discharge opioid consumption in the intervention vs. control group [14].

Rationale

Although there are many complete and ongoing studies assessing the efficacy of opioid-reduced/free protocols after TKA and THA, there is a scarcity of literature assessing patient preferences and perceptions regarding opioid use after surgery. Exploring patient beliefs and perceptions of opioid use can aid in identifying potential barriers, misconceptions, or fears that may affect patients' receptivity and adherence to opioid-sparing protocols or alternative pain management strategies. This information can guide the development of interventions to address these concerns, thereby optimizing patient outcomes. The primary objective of this study was to determine the proportion of patients who would be open to reducing or avoiding the use of opioid medications after TKA or THA. Secondary objectives involved identifying patient characteristics associated with receptivity to opioid-free or opioid-reduced postoperative care, determining patients’ perceptions regarding the efficacy and safety of opioids, and identifying potential barriers to opioid reduction or avoidance after TJA.

Please note that this study is part of an MSc thesis completed at McMaster University. This article was previously presented as a poster at the 2024 Canadian Orthopaedic Association (COA) Annual Meeting from June 12 to June 15, 2024.

## Materials and methods

Study design

We administered a cross-sectional survey and reported our findings according to the Consensus-Based Checklist for Reporting of Survey Studies (CROSS) [15]. 

Questionnaire development

Our questionnaire was developed with input from orthopaedic surgeons, epidemiologists, and experts in pain management. Our final survey included 36 items and five open-ended questions, and captured data on respondent characteristics, pain, surgical factors, openness to receiving post-surgical care without or with reduced opioids, perceptions regarding the efficacy and safety of opioids, and potential barriers to the implementation of an opioid-free or opioid-reduced postoperative care program. The open-ended questions asked participants to explain their receptivity to opioid-free or opioid-reduced postoperative care. We used a 0-100 mm visual analog scale (VAS) to measure patients’ pain severity. We qualified pain as mild (scores of ≤30), moderate (31-70), or severe (>70) [16]. We measured patients’ satisfaction with in-hospital and discharge medications using a 100-point scale (0: least satisfied, 100: most satisfied) [17].

Settings and participants

Participants were consecutively recruited from two orthopaedic clinics: St. Joseph’s Healthcare Hamilton and Juravinski Hospital, Hamilton, Ontario, Canada. To be included in the study, participants had to be aged 18 or over; presenting for a preoperative TJA appointment or a postoperative TJA appointment up to 12 months post-surgery; able to read, write, and understand English; and not judged to be cognitively impaired and able to complete the questionnaire. Participants were excluded if they were less than 18 years of age; not presenting for a preoperative TJA appointment or presenting for a postoperative TJA appointment more than 12 months post-surgery; not able to read, write, and understand English; and judged to be cognitively impaired and unable to complete the questionnaire.

Questionnaire administration

We collected data via a digital online survey hosted on Research Electronic Data Capture (REDCap) (https://www.project-redcap.org/) from February 2019 to February 2023 (recruitment was halted from the end of 2019 to the end of 2021, during the COVID-19 pandemic). A research assistant identified eligible patients in the two participating clinics. Eligible patients provided informed consent on a digital form on REDCap and completed the questionnaire on tablet computers, with an option to complete a paper version. All procedures were approved by the Hamilton Integrated Research Ethics Board (Project #5669).

Data analysis

We generated frequencies for all collected data and reported categorical data as proportions, and continuous data as means and standard deviations (SD) if normally distributed, or as medians and interquartile range (IQR) if not. We used the Shapiro-Wilk test to test for normality of continuous variables. We reported the overall prevalence of patients who would be open to receiving postoperative care without or with reduced opioids as the percentage of patients who responded “Definitely Yes” or “Probably Yes” to the corresponding questions.

We constructed two multivariable logistic regression analyses to evaluate factors associated with patients’ openness to receiving 1) opioid-free postoperative care, and 2) opioid-reduced postoperative care. We collapsed responses to create a binary dependent factor (“definitely yes” and “probably yes” vs. “unsure”, “probably no”, and “definitely no”). Guided by previous literature, we selected seven independent variables a priori that we felt may be associated with receptivity to opioid-reduced and opioid-free postoperative care and hypothesized the direction of association for each (Table 1) [9,18-24]. We calculated that a minimum of 70 respondents willing to avoid opioids and 70 willing to reduce opioid use after surgery would be required to ensure the reliability of our regression models (10 events for each independent variable considered) [25].

**Table 1:** Independent variables that may be associated with receptivity to opioid-reduced and opioid-free postoperative care and predicted direction of anticipated effects + indicates greater receptivity to opioid reduction or avoidance TJA: total joint arthroplasty

Variable	Description	Anticipated direction of openness
Highest education level	Respondent’s highest level of education achieved	Post-secondary qualification(s) or greater: (+) [14,20]
Adverse effects	Participants' perception of whether opioids have more or fewer side effects than other pain medications	Opioids have more side effects: (+) [16]
Opioids efficacy	Participants' perception of the efficacy of opioids in managing pain compared to other pain medications	Other medications work better or the same: (+) [16]
Other joint pain	Whether the patient has chronic pain in any other joint at the time of participation	No: (+) [17,18]
Current opioid use	Whether the patient is using opioids at the time of participation	No: (+) [19]
Timing of surgery	Whether the patient has already had their surgery or is scheduled for one	Patient has a planned/scheduled TJA: (+) [21]
Knee vs. hip replacement	Whether the patient has had or is scheduled for knee or hip replacement surgery	Hip replacement surgery: (+) [15]

We excluded respondents with missing data from our multivariable regression analyses using listwise deletion. Multicollinearity was deemed concerning if the variance inflation factor (VIF) for any independent factor was greater than five [26]. Additionally, we used the Hosmer-Lemeshow goodness-of-fit test to assess the overall fit of our models [27]. We reported measures of association as odds ratios (OR) and associated 95% confidence intervals (CI). We aimed to understand whether differences existed in patients' perceptions of opioids between those who had undergone surgery and those with a scheduled surgery. We used the Mann-Whitney U test to compare receptivity to opioid-free and opioid-reduced postoperative care, and perceptions of opioid efficacy and safety between patients with a scheduled surgery and those with a completed surgery. All comparisons were two-tailed, and an independent factor was considered statistically significant if it had a p-value <0.05 in the final multivariable model. We conducted all analyses using RStudio (v4.1.0; R Core Team 2021) [28], and we performed a summative content analysis to identify themes in responses to the open-ended questions [29].

## Results

Characteristics of respondents

We approached 200 patients to participate in our survey, of which 190 completed our questionnaire (95% response rate). All questions in this questionnaire were optional, with some participants choosing not to answer certain questions, resulting in a variable denominator within the responses. Most respondents (n=149; 78%) had undergone surgery, and 22% (n=41) were scheduled for knee/hip arthroplasty. Among patients who had already undergone surgery, 46% (n=67) completed the survey one to three months postoperatively. The median age of participants was 67 years (IQR: 60-73), and 65% (n=123) were women (Table 2).

**Table 2:** Baseline demographics of patients ^1^N=180; ^2^N=187 (since not all respondents answered every question, the denominator varies for each question) IQR: interquartile range; THA: total hip arthroplasty; TJA: total joint arthroplasty; TKA: total knee arthroplasty

Variables	Values
Age, years^1^, median (IQR)	67 (60, 73)
Gender, n (%)	
Male	66/189 (35%)
Female	123/189 (65%)
Education level, n (%)	
No high school	7/188 (4%)
Some high school	18/188 (10%)
High school completed	46/188 (24%)
College/trade school	67/188 (36%)
Bachelor's degree	35/188 (19%)
Graduate degree	15/188 (8%)
Income, n (%)	
≤$20,000	14/171 (8%)
$20,001-$40,000	32/171 (19%)
$40,001-$60,000	32/171 (19%)
$60,001-$80,000	38/171 (22%)
$80,001-$100,000	25/171 (15%)
>$100,001	30/171 (18%)
Knee vs. hip replacement, n (%)	
TKA	164/189 (87%)
THA	25/189 (13%)
Level of joint pain today^2^,median (IQR)	25 (6, 52)
Duration of joint pain, years, n (%)	
0-1	12/98 (12%)
2-3	26/98 (27%)
4-5	18/98 (18%)
6+	42/98 (43%)
Other joint pain, n (%)	110/188 (59%)
Currently using pain medications, n (%)	141/189 (75%)
Currently using opioids, n (%)	38/190 (20%)
Surgery scheduled or completed, n (%)	
TJA scheduled	41/190 (22%)
TJA completed	149/190 (78%)

Of note, 43% (n=42) of participants indicated that they had knee/hip joint pain for six or more years before receiving or being scheduled for surgery, and 59% (n=110) were experiencing chronic pain in joints other than the one targeted for replacement; 24% (n=45) of individuals reported using prescription opioids at the time of survey completion (Table 3). Most participants completing the questionnaire postoperatively reported mild current knee/hip pain (median: 20 mm on a 100 mm VAS; IQR: 2-50) (Table 4), while those awaiting surgery typically indicated a moderate level of pain (median: 60 mm on a 100 mm VAS; IQR: 49-80) (Table 5). 

**Table 3:** Other reported locations of joint pain and prescription and non-prescription medication use Since not all respondents answered every question, the denominator varies for each question

Characteristics	N (%)
Other joint pain	110/188 (59%)
Other knee	65/188 (35%)
Other hip	3/188 (2%)
Knee(s)	5/188 (3%)
Hip(s)	23/188 (12%)
Ankle(s)	11/188 (6%)
Shoulder(s)	19/188 (10%)
Elbow(s)	4/188 (2%)
Wrist(s)	8/188 (4%)
Hand(s)	13/188 (7%)
Neck	10/188 (5%)
Back	27/188 (14%)
Other	6/188 (3%)
Currently using pain medications	141/189 (75%)
Naproxen	17/189 (9%)
Toradol	5/189 (3%)
Celecoxib	14/189 (7%)
Diclofenac	17/189 (9%)
Indomethacin	1/189 (0.5%)
Meloxicam	3/189 (2%)
Codeine	9/189 (5%)
Morphine	9/189 (5%)
Oxycodone	8/189 (4%)
Hydromorphone	19/189 (10%)
Fentanyl	0/189 (0%)
Gabapentin	5/189 (3%)
Pregabalin	3/189 (2%)
Amitriptyline	0/189 (0%)
Cannabis	6/189 (3%)
Ibuprofen	24/189 (13%)
Aspirin	5/189 (3%)
Acetaminophen	83/189 (44%)
I don't remember	5/189 (3%)
Others	9/189 (5%)

**Table 4:** Characteristics and perceptions of patients whose surgery completed in the past year ^1^N=142; ^2^N=146; ^3^N=148; ^4^N=145; ^5^N=144 (since not all respondents answered every question, the denominator varies for each question) IQR: interquartile range; VAS: visual analog scale

Variables	Values
Age^1^, years, median (IQR)	66 (60, 73)
Gender, n (%)	
Male	47/148 (32%)
Female	101/148 (68%)
Level of joint pain today (100mm VAS)^2^,median (IQR)	20mm (2, 50)
Duration since surgery, months, n (%)	
1	19/145 (13%)
1-3	67/145 (46%)
3-6	20/145 (14%)
6-12	39/145 (27%)
Number of joints replaced, n (%)	
1 joint	106/147 (72%)
2 joints at different times	29/147 (20%)
2 joints simultaneously	12/147 (8%)
Current pain vs. pre-op pain, n (%)	
Much better	96/147 (65%)
A bit better	26/147 (18%)
About the same	12/147 (8%)
A bit worse	8/147 (5%)
A lot worse	5/147 (3%)
Pain level in the first few days post-op^3^,median (IQR)	75 (52, 90)
Satisfaction with in-hospital medications^4^,median (IQR)	90 (75, 100)
Satisfaction with medications on discharge^5^,median (IQR)	84 (58, 99)

**Table 5:** Characteristics and perceptions of patients with total joint arthroplasty surgery scheduled ^1^N=38; ^2^N=41; ^3^N=40 (since not all respondents answered every question, the denominator varies for each question) IQR: interquartile range; VAS: visual analog scale

Variables	Values
Age^1^, years, median (IQR)	67 (58, 73)
Gender, n (%)	
Male	19/41 (46%)
Female	22/41 (54%)
Level of joint pain today (VAS)^2^,median (IQR)	60 (49, 80)
Time till surgery, months, n (%)	
<1	13/41 (32%)
1-4	3/41 (7%)
4-8	4/41 (10%)
8-12	1/41 (2%)
12+	20/41 (49%)
Not sure	0/41 (0%)
Number of joints to be replaced, n (%)	
1 joint	34/41 (83%)
2 joints at different times	6/41 (15%)
2 joints simultaneously	1/41 (2%)
Post-op pain expectations (VAS)^3^,median (IQR)	80 (70, 88)

Perceptions of opioid efficacy and adverse effects

Of note, 75% (123/165) of respondents perceived that opioids were more effective than other analgesics for managing pain, and 68% (112/164) believed that opioids had more side effects than other pain medications (Table 6). There was no statistically significant difference between the preoperative and postoperative groups for perceptions of opioid efficacy; however, 33% (43/130) of post-surgical patients believed that opioids and other pain medications had a similar number of side effects compared to 15% (5/34) of patients scheduled for surgery (p<0.05). Of participants who had undergone surgery, 44% (66/149) experienced constipation, 35% (52/149) had drowsiness, and 32% (48/149) had no side effects from any of their pain medications (Table 7). When asked to select one harm of opioids they were most concerned with, most participants endorsed addiction (43%; 75/175), while 21% were not concerned about the adverse effects of opioids (Table 8). When asked to indicate their level of concern across nine harms associated with opioids, most respondents were concerned about constipation (53%), and least concerned about accidental overdose (25%; 23/91) or death (22%; 20/91) (Table 9).

**Table 6:** Perceptions of opioid efficacy and safety, and receptivity to opioid-reduced/free postoperative care Since not all respondents answered every question, the denominator varies for each question PMs: pain medications

Perception	Surgery planned, n (%)	Surgery complete, n (%)	Overall, n (%)
Efficacy of opioids vs. other PMs			
Opioids work much better	13/33 (39)	62/132 (47)	75/165 (45)
Opioids work a bit better	12/33 (36)	36/132 (27)	48/165 (29)
They work about the same	5/33 (15)	24/132 (18)	29/165 (18)
Other PMs work a bit better	25/33 (6)	4/132 (3)	6/165 (4)
Other PMs work much better	15/33 (3)	6/132 (5)	7/165 (4)
Side effects of opioids vs. other PMs			
Opioids have many more side effects	18/34 (53)	38/130 (29)	56/164 (34)
Opioids have a few more side effects	10/34 (29)	46/130 (35)	56/164 (34)
Side effects are about the same	5/34 (15)	43/130 (33)	48/164 (29)
Other PMs have a few more side effects	1/34 (3)	1/130 (1)	2/164 (1)
Other PMs have many more side effects	0/34 (0)	2/130 (2)	2/164 (1)
Receptivity to opioid-free post-op care			
Definitely yes	9/41 (22)	31/146 (21)	40/187 (21)
Probably yes	9/41 (22)	25/146 (17)	34/187 (18)
Not sure	15/41 (37)	14/146 (10)	29/187 (16)
Probably no	5/41 (12)	20/146 (14)	25/187 (13)
Definitely no	3/41 (7)	56/146 (38)	59/187 (32)
Receptivity to opioid-reduced post-op care			
Definitely yes	10/35 (29)	36/147 (24)	46/182 (25)
Probably yes	7/35 (20)	38/147 (26)	45/182 (25)
Not sure	15/35 (43)	22/147 (15)	37/182 (20)
Probably no	1/35 (3)	10/147 (7)	11/182 (6)
Definitely no	2/35 (6)	41/147 (28)	43/182 (24)

**Table 7:** Adverse effects to any pain medications among patients who had undergone total joint arthroplasty

Adverse effect	N (%), (N=149)
Constipation	66 (44%)
Drowsiness	52 (35%)
No side effects	48 (32%)
Inadequate pain control	24 (16%)
Nausea/vomiting	22 (15%)
Lack of coordination	17 (11%)
Accidental overdose	1 (1%)
Addiction	1 (1%)
Liver toxicity	0 (0%)

**Table 8:** Side effects of opioids that patients were most concerned about in relation to non-opioid analgesics

Characteristic	N (%), (N=175)
Addiction	75 (43%)
Not concerned about any side effects	36 (21%)
Constipation	22 (13%)
Liver toxicity	11 (6%)
Nausea/vomiting	9 (5%)
Lack of coordination	5 (3%)
Death	5 (3%)
Drowsiness	4 (2%)
Inadequate pain control	3 (2%)
Other	3 (2%)
Accidental overdose	2 (1%)

**Table 9:** Patients’ level of concern about the adverse effects of opioids

Characteristic	Not concerned at all, n (%)	Not really concerned, n (%)	Neither concerned nor unconcerned, n (%)	Concerned, n (%)	Very concerned, n (%)
Addiction	36/93 (39%)	10/93 (11%)	6/93 (6.5%)	19/93 (20%)	22/93 (24%)
Nausea/vomiting	39/93 (42%)	21/93 (23%)	11/93 (12%)	15/93 (16%)	7/93 (7.5%)
Constipation	17/92 (18%)	11/92 (12%)	15/92 (16%)	34/92 (37%)	15/92 (16%)
Drowsiness	32/93 (34%)	19/93 (20%)	17/93 (18%)	22/93 (24%)	3/93 (3.2%)
Lack of coordination	38/92 (41%)	16/92 (17%)	17/92 (18%)	17/92 (18%)	4/92 (4.3%)
Inadequate pain control	41/90 (46%)	14/90 (16%)	7/90 (7.8%)	18/90 (20%)	10/90 (11%)
Liver toxicity	38/93 (41%)	11/93 (12%)	10/93 (11%)	21/93 (23%)	13/93 (14%)
Accidental overdose	49/91 (54%)	12/91 (13%)	7/91 (7.7%)	13/91 (14%)	10/91 (11%)
Death	54/91 (59%)	7/91 (7.7%)	10/91 (11%)	7/91 (7.7%)	13/91 (14%)

Receptivity to opioid-free and opioid-reduced postoperative care

It was found that 40% (74/187) of participants were receptive to opioid-free postoperative care, and 50% (91/182) were open to opioid-reduced postoperative care. More participants indicated that they would definitely not be open to receiving opioid-free care (32%; 59/187) compared to opioid-reduced care (24%; 43/182). There was no significant difference between the planned and completed surgery groups regarding receptivity to opioid-free (44% vs. 38%) or opioid-reduced care (49% vs. 50%). However, most participants who were scheduled for surgery were unsure if they would be open to opioid elimination (37%, 15/41) or reduction (43%, 15/35), whereas after surgery, a much smaller proportion were uncertain and a larger proportion was unwilling to reduce or avoid opioids (Table 6). 

In our final adjusted regression models, the odds of being receptive to opioid-free postoperative care were greater for those who believed that other medications were similarly or more effective than opioids (OR: 3.4; 95% CI: 1.4-8.3), and for those who perceived that opioids have more side effects than non-opioid analgesia (OR: 4.3; 95% CI: 1.8-11.9) (Table 10).

**Table 10:** Factors associated with openness to opioid avoidance after surgery ^*^P<0.05 (A Wald test was used to assess whether the p-value is significant or not) CI: confidence interval; OR: odds ratio; THA: total hip arthroplasty; TJA: total joint arthroplasty; TKA: total knee arthroplasty

Predictor	OR	95% CI	P-value
Adverse effects			
Other medications have the same or more side effects	Ref	—	
Opioids have more side effects	4.34	1.76, 11.9	0.002^*^
Opioids' efficacy			
Opioids work better	Ref	—	
Other medications work better or the same	3.39	1.44, 8.33	0.006^*^
Surgery planned or completed			
TJA planned/scheduled	Ref	—	
TJA completed	0.90	0.37, 2.26	0.8
Highest level of education			
Less than high school	Ref	—	
High school graduate	2.79	0.73, 12.5	0.15
Post-secondary qualification(s)	1.58	0.46, 6.50	0.5
Other joint pain			
No	Ref	—	
Yes	0.69	0.32, 1.48	0.3
Current opioid use			
Yes	Ref	—	
No	2.46	0.93, 7.24	0.081
Knee vs. hip replacement			
TKA	Ref	—	
THA	1.84	0.65, 5.19	0.2

The odds of being open to opioid-reduced surgery were greater for patients who believed that opioids have more side effects than non-opioids (OR: 3.4; 95% CI: 1.5-7.9) and for those who did not report using opioids at the time of the survey completion (OR: 2.5; 95% CI: 1.04-6.4) (Table 11). The VIFs were less than two for each independent variable in both models, suggesting no issues with multicollinearity. The Hosmer-Lemeshow test demonstrated a good fit for both models.

**Table 11:** Factors associated with openness to opioid reduction after surgery ^*^P<0.05 (A Wald test was used to assess whether the p-value is significant or not) CI: confidence interval; OR: odds ratio; THA: total hip arthroplasty; TJA: total joint arthroplasty; TKA: total knee arthroplasty

Predictor	OR	95% CI	P-value
Adverse effects			
Other medications have the same or more side effects	Ref	—	
Opioids have more side effects	3.40	1.53, 7.91	0.003^*^
Opioids' efficacy			
Opioids work better	Ref	—	
Other medications work better or the same	2.00	0.85, 4.88	0.12
Surgery planned or completed			
TJA planned/scheduled	Ref	—	
TJA completed	1.22	0.46, 3.22	0.7
Highest level of education			
Less than high school	Ref	—	
High school graduate	2.58	0.77, 9.23	0.13
Post-secondary qualification(s)	1.20	0.40, 3.75	0.7
Other joint pain			
No	Ref	—	
Yes	0.67	0.31, 1.40	0.3
Current opioid use			
Yes	Ref	—	
No	2.50	1.04, 6.35	0.045^*^
Knee vs. hip replacement			
TKA	Ref	—	
THA	1.76	0.65, 4.99	0.3

Qualitative findings

In the questions with open responses, 114 and 87 participants provided reasons for their level of openness to opioid-free and opioid-reduced care, respectively (Tables 12, 13). Most participants who were receptive to opioid elimination (42%; 15/36) or reduction (48%; 19/40) after surgery had previously experienced or were concerned with the adverse effects of opioids (e.g., constipation, addiction, falls). Of note, 62% (48/78) of participants who were not open to postoperative opioid elimination and 49% (23/47) who were unreceptive to opioid reduction believed that they could not tolerate pain without, or with less, opioids. Participants, regardless of their willingness for opioid reduction/elimination, were uncertain of non-opioid alternatives and whether they would be effective in managing their pain. Respondents who were hesitant about opioid-free/reduced care also believed that opioids were needed for better sleep, mobility, and rehabilitation (Table 14).

**Table 12:** Themes and sub-themes of patient responses to reasons for openness to opioid avoidance

Themes and sub-themes	Frequency of endorsement, n (%)	Examples of patient responses
Open to receiving opioid-free postoperative care (n=36/74)		
Experienced or concerned about the adverse effects of opioids	15 (42%)	“To avoid constipation, and also pain was not that bad.” “Don't like the side effect.”
Opioids lack efficacy in managing pain	2 (6%)	“[Opioids] did not work for me.” “Unable to tolerate morphine.”
Does not want/need to use opioids for managing pain	13 (36%)	“Hardly used any of the meds.” “Don't like to take really any drugs for pain.” “Do not want them in my body.”
Knowledge gaps		
Pain expectations after surgery	1 (3%)	“I didn't know what to expect.”
Efficacy of non-opioid alternatives	2 (6%)	“If there is a 'good' alternative, it makes sense not to be reliant on opioids.” “Yes, as long as they were effective.”
Satisfied with non-opioid alternatives	3 (8%)	“Patient is okay with just Tylenol.”
Unable to monitor rehabilitation (pain is masked)	1 (3%)	“Rehab could be compromised if the pain is masked, also, I'm not a fan of opioids.”
Not open to receiving opioid-free postoperative care/not sure (n=78/113)		
Intolerable level of pain without opioids	48 (62%)	“Can't tolerate the pain without.” “Pain is unbearable.”
Faster and better pain relief with opioids	6 (8%)	“Very good pain relief from opioids, and there was a lot of pain after surgery. Morphine helped more, but didn't receive diff doses.”
Knowledge gaps		
Efficacy of non-opioid alternatives	11 (14%)	“Not sure what pain level would be like without them.” “I don't know that there is anything that would work as well.”
Strategies to manage pain immediately after discharge	1 (1%)	“Need better info on immediate pain meds requirements after 72 hours.”
Weaning off opioids after use	1 (1%)	“Helpful if they are only given opioids for a little time, and help with getting off of them. Hard to get off hydrocodone even after one week, and it causes many side effects. Need something to naturally and gently stop opioid use.”
Concerned about adverse effects	3 (4%)	“The pain was horrendous, and I was conscious of the addictive nature.” “Fear of addiction and mental illness.”
Expectation of being pain-free	7 (9%)	“Not enough pain management.” “Don't want pain.”
Need opioids for better sleep, increased mobility, and rehabilitation	5 (6%)	“Really needed them for the pain and to do the exercises.” “Physiotherapy is hard and it pushes patients, and they would be in extreme pain after physio.”
Positive experience/outcomes after past opioid use	5 (6%)	“I know they are effective after surgery from my past experiences. I don't want to try something that may not work.”

**Table 13:** Themes and sub-themes of patient responses to reasons for openness to opioid reduction

Themes and sub-themes	Frequency of endorsement, n (%)	Examples of patient responses
Open to receiving opioid-reduced postoperative care (n=40/91)		
Experienced or concerned about the adverse effects of opioids	19 (48%)	“Don't want to be hooked.” “Find that medication changes your personality and can be addictive.”
Pain is/was manageable with fewer opioids	10 (25%)	“Hardly used any of the meds.” “Pain was manageable without opioids as time went on.”
Lack of knowledge about the efficacy of non-opioid alternatives	3 (8%)	“Need to know what it is, and how effective it is.”
Reduced opioids allow for mobility and rehabilitation	2 (5%)	“It's definitely hard to do physio without pain meds.” “So, I can walk.”
Cannot tolerate pain with no opioids	3 (8%)	“To control pain but lessen the risk of addiction.” “Pain was intense, don't want to suffer.”
Does not want/need to use opioids for managing pain	4 (10%)	“Do not want them.” “Prefer no opioids.”
Not open to receiving opioid-reduced postoperative care/not sure (n=47/91)		
Need more opioids to control/tolerate pain	23 (49%)	“Because if anything, I need stronger pain medication.” “Pain is too intense.”
Already using a low dose/amount	3 (6%)	“Already low dosage would not like any lower.” “Taking the minimum now.”
Lack of knowledge about the efficacy of non-opioid alternatives	9 (19%)	“Depends on whether the substitutes control the pain as well.” “Only if I was ASSURED that they would work effectively.”
Expectation of being pain-free	4 (9%)	“I want pain gone.” “Pain wasn't taken away initially.”
Dissatisfied with pain management medications	1 (2%)	“Medication was not as effective as the previous surgery.”
Need more opioids for increased mobility and rehabilitation	5 (11%)	“Needed medication for exercise and physio want more, not less.” “If you have to do everything yourself, you need meds.”

**Table 14:** Themes and sub-themes of patients’ additional comments and thoughts

Themes and sub-themes	Frequency of endorsement, n (%) (n=46/190)	Examples of patient responses
Experienced or concerned about adverse effects	11 (24%)	“They worked, just that the side effects were strong and needed to be taken off. Addiction was at the forefront of the mind to decrease the dosage.” “Opioids make me feel 'very off,' like I wasn't myself. If another option provided the same pain relief, I would be very willing to try it.”
Many opioids were left over/did not use any opioids	4 (9%)	“Hardly used the medications and for a short time.” “I took the prescribed opiates for a few weeks, and then just went to Tylenol.”
Cannot tolerate pain without opioids	4 (9%)	“Opioid control is essential during the immediate post-op period, 24-72 hrs. After that, non-opioids are quite adequate.” “When the nurse comes and asks what pain it is, do not say you do not have any, since they give you none. When the block wears off, you are in so much pain that it becomes difficult to get rid of the pain afterwards.”
Has not had negative experiences/outcomes with opioids	1 (2%)	“Didn't have any negative experiences with opioids, and they should give them to people who need them, but careful screening is needed before they give them.”
Lack of patient autonomy in pain management	2 (4%)	“I believe that it is a patient's decision whether the risks and side effects outweigh the benefits…”, “…I think it is important to look at a patient as an individual.”
Surgeons' restrictions vs. patient needs	3 (7%)	“I need a higher dose, and doctors are too reluctant to prescribe. It angers me to no end!” “I understand that doctors worry about addiction; however, some patients, such as myself, need proper pain control, and unfortunately for me, hydromorphone did not help me.”
Satisfied with surgery, fewer opioids, and outcomes	5 (11%)	“Tylenol worked for me instead of opioids. ”I was very pleased with my surgery, and I made sure that I did my exercises that I was taught at physio for the time that I was waiting for surgery, and I made sure I did everything I was told to do after surgery also.”
Need for safe and effective alternatives	6 (13%)	“I wish there were a strong medication for pain that does not cause nausea, dizziness, + general malaise. The side effects of hydromorphone are awful.” “Need a better plan rather than opioids. and it needs to be something that's not addictive or that aggressive…”
Need for better strategies to wean off opioids	1 (2%)	“Need a gentler method for patients to get off of opioids after they are done with their prescription, so it's not so abrupt.”
Opioids needed for better sleep, mobility, and rehabilitation	9 (20%)	“Other than knee surgery - I used to have Percocet handy for chronic knee pain - bad for constipation, but very good for pain management. When I took a dose, I could get a lot of work done as opposed to sitting around moaning and groaning. I was not addicted, but it was like having a holiday from the pain.”
Increased monitoring needed to decrease risk of side effects	4 (9%)	“When managed properly, side effects can be avoided.” “Most of the side effects occur if the meds aren't taken properly.”
Opioids are more effective than alternatives for pain control	1 (2%)	“Opioids help more than any other pain medications I’ve tried…”
Opioids are unnecessary/should be banned	4 (9%)	“Overall, I don't take any medications, so I prefer to stay off everything.” “Tell the doctor to stop giving it to patients.” “Should be banned.”

## Discussion

Overall, 40% of participants reported a willingness to receive post-TJA care without opioids, and 50% with reduced opioids, regardless of whether they were awaiting or had undergone surgery. Receptivity was associated with participants’ perceptions of the safety and efficacy of opioids compared to other pain medications, and present opioid use. Unwillingness to pursue opioid-free postoperative care was associated with surgical status; most patients awaiting surgery were uncertain, while those who had already undergone surgery were less unsure and more likely to be unwilling to forgo or reduce opioid use.

Of note, 74% of respondents believed that opioids were more effective for managing pain compared to non-opioid analgesics, and those currently using opioids were most enthusiastic. Similarly, a cross-sectional study of 715 adults attending an emergency department (ED) reported that 88% who had previously used opioids found them effective at relieving pain, and 63% would take them again; experiencing adverse effects was associated with reduced willingness to use opioids again (OR: 0.70; 95% CI: 0.66-0.75) [20]. Our multivariable analyses revealed that patients who believed that alternative medications were equally or more effective than opioids were more likely to be receptive to opioid-free postoperative care but not opioid-reduced care. This preference may be driven by concerns about the potential side effects of opioids, as our qualitative analysis found that those patients who had experienced adverse effects of opioids were most willing to avoid them. 

We found that 31% of participants believed that other non-opioid pain medications had a similar or greater number of side effects compared to opioids. However, a 2022 systematic review, comparing postoperative opioid prescribing with opioid-free analgesia, found that opioid prescribing was associated with an increased risk of vomiting, nausea, constipation, dizziness, and drowsiness [30]. Similarly, a subsequent randomized controlled trial, evaluating the effect of a multimodal, opioid-sparing postoperative pain management approach compared with the current standard of care, in 200 patients undergoing arthroscopic shoulder or knee surgery, found that more patients reported medication-related adverse effects in the standard care group (32% vs 19%, p=0.048) [11].

Our respondents expressed significant concerns about opioid-related constipation, but showed considerably fewer concerns about the risks of overdose or death. Opioid-induced constipation is common among patients prescribed opioids for chronic pain, and a systematic review of patients’ values and preferences found high concerns with the gastrointestinal effects of opioids [31,32]. The absolute risk of non-fatal opioid overdose is 0.2%, and the risk of death is 0.1% following prescription for chronic pain; also, a prior focus group study of pain patients found that they placed little importance on the small likelihood of accidental overdose or death [33,34]. 

Patients who were using opioids at the time of survey administration showed greater reluctance to reduce opioid use after surgery. Furthermore, our qualitative analysis found that most patients who were unreceptive to opioid-free/reduced care expected intolerable or severe postoperative pain requiring opioid analgesics. These findings are consistent with a qualitative study, including 11 opioid-naive patients undergoing elective knee arthroscopy, which reported that participants’ attitudes toward postoperative pain and opioids were influenced by perceived tolerance to pain based on personal experience [24]. A prospective cohort study of 259 surgical patients found that patients with a high self-perceived pain tolerance may be better prepared to manage post-surgical pain [34]. Conversely, those who endorsed low pain tolerance experienced more anxiety regarding their ability to manage pain [24,34]. Heightened anxiety about pain and catastrophic thinking in the preoperative period may also increase postoperative pain intensity [35-37]. Patients with prior positive experiences with opioids or with greater concerns about postoperative pain are more likely to desire opioids after surgery. Postoperative pain is a primary concern for up to 80% of surgical patients, and preoperative counseling and education may improve patients' knowledge and ease their anxieties surrounding postoperative pain management [38].

While preoperative education is already a fundamental element of patient care, a significant number of patients expressed hesitancy to opioid reduction or elimination and held misconceptions, emphasizing the necessity for a more targeted and effective approach in educating patients about appropriate expectations of pain control, the risks and benefits of opioid pharmacotherapy, and alternatives. Although each patient is unique, identifying common trends and misconceptions in patient beliefs regarding opioid use may allow physicians to anticipate and proactively address these issues. This approach may lead to more effective education, ultimately improving shared decision-making processes. It is important to recognize that preoperative education extends beyond merely providing information; it must empower patients with knowledge that aligns with their preferences and concerns.

Limitations

Our study has several limitations. Our sample primarily consisted of elderly men who had undergone TKA, whose data were obtained from two clinics in Hamilton, ON, and the generalizability of findings to other populations and regions is uncertain. Furthermore, our study may have been impacted by recall bias as some participants had undergone surgery up to one year before the survey, and social desirability bias may have affected their responses. However, surveys were anonymous to reduce the possibility of social desirability bias. Also, the study did not assess the actual opioid prescribing practices of healthcare providers or the outcomes of patients who chose to reduce or avoid opioids after surgery, which limits the ability to draw conclusions about the impact of patient receptivity on clinical outcomes. Future studies could consider the potential influence of healthcare providers' attitudes and beliefs towards opioid prescribing, as provider-related factors may play a significant role in shaping patient behavior and decision-making.

## Conclusions

Many joint arthroplasty patients were willing to reduce or avoid the use of postoperative opioids, and receptivity was strongly associated with beliefs regarding the comparative benefits and harms of alternatives. Our findings point to opportunities to reduce the use of opioids after TJA and provide valuable insights into the baseline perceptions of patients towards opioid reduction and avoidance after surgery. The identified common themes and misconceptions may inform the development of more targeted communication and patient education strategies, ultimately improving patient engagement and adherence to recommended pain management plans.
